# From linoleic acid to hexanal and hexanol by whole cell catalysis with a lipoxygenase, hydroperoxide lyase and reductase cascade in *Komagataella phaffii*

**DOI:** 10.3389/fmolb.2022.965315

**Published:** 2022-12-12

**Authors:** Chiam Hashem, Julius Hochrinner, Moritz B. Bürgler, Claudia Rinnofner, Harald Pichler, Margit Winkler

**Affiliations:** ^1^ Institute of Molecular Biotechnology, TU Graz, NAWI Graz, Graz, Austria; ^2^ Austrian Centre of Industrial Biotechnology (acib GmbH), Graz, Austria; ^3^ BioTechMed Graz, Graz, Austria

**Keywords:** lipoxygenase (LOX), hydroperoxide lyase (HPL), fatty acid, *Komagataella phaffii (P. pastoris)*, green leaf volatiles (GLVs)

## Abstract

Green leaf volatiles (GLVs) cover a group of mainly C6-and C9-aldehydes, -alcohols and -esters. Their name refers to their characteristic herbal and fruity scent, which is similar to that of freshly cut grass or vegetables. Lipoxygenases (LOXs) catalyze the peroxidation of unsaturated fatty acids. The resulting hydroperoxy fatty acids are then cleaved into aldehydes and oxo acids by fatty acid hydroperoxide lyases (HPLs). Herein, we equipped the yeast *Komagataella phaffii* with recombinant genes coding for LOX and HPL, to serve as a biocatalyst for GLV production. We expressed the well-known 13*S*-specific LOX gene from *Pleurotus sapidus* and a compatible HPL gene from *Medicago truncatula*. In bioconversions, glycerol induced strains formed 12.9 mM hexanal using whole cells, and 8 mM hexanol was produced with whole cells induced by methanol. We applied various inducible and constitutive promoters in bidirectional systems to influence the final ratio of LOX and HPL proteins. By implementing these recombinant enzymes in *Komagataella phaffii*, challenges such as biocatalyst supply and lack of product specificity can finally be overcome.

## Introduction

Plants generate a variety of volatile organic compounds that act as hormones, performing multiple functions, e.g. in plant defense, communication and stress responses. These compounds are used in plant and insect control as well as in the food and cosmetic industries. Green leaf volatiles, in particular, are widely known as fragrances and flavors for their refreshing smell of grass, fruits and vegetable scents (Holopainen and Blande, 2012; Meena et al., 2017; Li et al., 2021).

A biocatalytic two step cascade to GLV production attracted our attention. The first step requires the oxidation of polyunsaturated fatty acids to produce oxylipins (Matsui, 2006; Sarang et al., 2021). This is catalyzed by lipoxygenases (LOXs, EC 1.13.11). LOXs are metal ligand biocatalysts, with either a non-heme iron or manganese center, that act on polyunsaturated fatty acids, e.g. linoleic acid, to yield various important oxylipins, such as the hydroperoxy octadecadienoic acid (HPODE). Oxylipins can then be converted by hydroperoxide lyases (HPLs, EC 4.1.2.92) to yield GLV aldehydes and ω-oxo acids as a co-product. HPLs are heme iron biocatalysts, belonging to a unique family of cytochrome P450 enzymes, which do not operate as monooxygenases. In contrast to classical cytochrome P450 enzymes, HPLs require neither oxygen nor NADPH-dependent reductases (Matsui et al., 1996; Ivanov et al., 2005; D’Auria et al., 2007; Haeggström and Funk, 2011; Wennman et al., 2015; Stolterfoht et al., 2019; Vincenti et al., 2019; Shi et al., 2020).

Plant extracts have frequently been employed as sources of LOX and HPL in the generation of GLVs so far. Major drawbacks of crude plant extracts, however, are unpredictable enzyme amounts with strong seasonal variations, land use for catalyst growth, high viscosity, challenging downstream processing and (iso)enzymes in the mixture. The presence of plant lipids and various other enzymes in biological samples lead to mixed product formation. Low yield of desired products result from relatively poor conversion rates of HPLs (Rabetafika et al., 2008; Gigot et al., 2010). From an economic and environmental point of view, the synthesis of green leaf volatiles using microorganisms such as yeast or bacteria in bioreactors is an attractive alternative (Cheetham, 1997). Jacopini et al. (2016) introduced a recombinant olive HPL into *Escherichia coli* to produce C-6 aldehydes. Their most efficient approach allowed them to produce around 5.61 mM of hexanal, deriving from HPODE as starting material. In 2004 and 2009, Bourel et al. (2004); Santiago-Gómez et al. (2009) produced C-6 aldehydes with the green bell pepper HPL in the yeast *Yarrowia lipolytica*. However, highest yields reported were around 3.5 mM and 6 mM, respectively. Another approach was reported by Buchhaupt et al. (2012), in which a combination of LOX and HPL was employed to produce GLVs in *Saccharomyces cerevisiae*. The highest yield of GLV was estimated to be around 0.6 mM, however, biotransformation activity stalled after about 90 min.

The use of recombinant enzymes, produced in the methylotrophic yeast *Komagataella phaffii,* has led to an improvement in the flavor and fragrance production. The eukaryotic organism reaches high cell densities during growth and facile engineering of stable strains by genomic integration is one of the many advantages of using *K. phaffii* as a host (Gasser et al., 2013; Bill, 2014). Furthermore, multi-gene expression in *K. phaffii*, as introduced by Vogl et al. (2018a), made the yeast species particularly appealing for fine-tuning co-expression of two genes in one host. Conventional monodirectional promoters were compared to sets of bidirectional promoters, revealing improved capabilities towards multi-gene expression after screening a library of synthetic bidirectional promoters.

**SCHEME 1:**
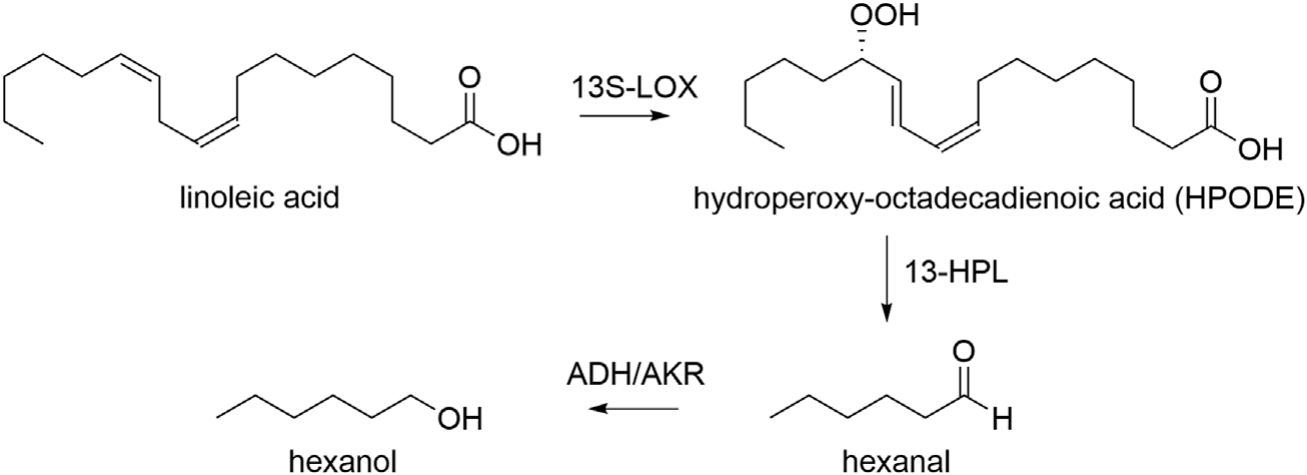
Transformation of linoleic acid to GLVs hexanal and hexanol. 13*S*-LOX: recombinant lipoxygenase, 13-HPL: recombinant hydroperoxide lyase, ADH: endogenous alcohol dehydrogenase, AKR: endogenous aldo-keto reductase.

In this work, we use *K. phaffii* to express the LOX of *Pleurotus sapidus*, and HPL from *Medicago truncatula/sativa* (Noordermeer et al., 2000; Hughes et al., 2006; Kelle et al., 2014). We used bidirectional, pairs of several inducible and constitutive promoters to control the ratio of the two proteins, as well as to be able to induce gene expression with both, methanol or glycerol. Both enzyme classes are essential in our approach for transforming linoleic acid into the green leaf volatile hexanal and oxo-dodecenoic acid. One molecule of linoleic acid is transformed into one molecule aldehyde, which may subsequently be reduced to hexanol by intracellular enzymes i.e. alcohol dehydrogenases, yielding one of two GLVs, hexanal or hexanol (Scheme 1) (Bate et al., 1998; Salas and Sánchez, 1998).

## Materials and methods

### General

Standard reagents were obtained from Sigma-Aldrich (Vienna, Austria) or Roth GmbH & Co. KG (Karlsruhe, Germany) with the highest purity available. Restriction enzymes were obtained from Thermo Scientific (St. Leon Rot, Germany). Bacto™ peptone, Bacto™ yeast extract and Difco™ yeast nitrogen base (YNB) were obtained from Becton, Dickinson and Company (Schwechat, Austria). Zeocin™ was purchased from InvivoGen (Vienna, Austria) and linoleic acid was purchased from Sigma-Aldrich (≥ 99%, CAS: 60-33-3).

### Vector and strain construction

Genes and fragments were codon optimized using a high methanol codon usage table for *K. phaffii* (Abad et al., 2010) and ordered at Twist Bioscience (South San Francisco, United States). LOX genes with an *N*-terminal His_6_-tag were amplified from two Twist fragments each, with the primers listed in Table 1. For example, the upstream sequence of *Ps*LOX was amplified with primers pBSY3Z_fw and PsLOX1rev and the downstream sequence with PsLOX2fw and pBSY3Z_rv, respectively. The vector backbones pBSY3Z for intracellular expression and pBSY3S1Z for protein secretion, respectively, were prepared by double restriction digest of pBSY3Z or pBSY3s1Z vector (Rieder et al., 2021) preparations by *Sap*I. Gene fragments were assembled with the vector backbone by the Gibson method. Similarly, single gene fragments of HPL genes with an *N*-terminal His_6_-tag were assembled to the vector backbone.

**TABLE 1:** Primer list and sequences used for sequencing and construction of vectors.

Primer name	Primer sequence (5′-3′)	Primer name	Primer sequence (5′-3′)
Seq_Pucfw	CCT​GGT​ATC​TTT​ATA​GTC​CTG​TCG	*Ps*LOX2fw	CAA​CAA​GAG​AAC​CAA​CCC​ATT​AGA​C
Seq_PCatfw	GCA​ATT​AAC​TTC​CCT​CGA​TTG​G	*Pa*LOX1rv	CAG​TAC​CAG​TAA​GCA​GTT​TAT​CTA​CG
Seq_ILV5_rv	GAT​TTA​TGC​CAG​TCT​TGG​TCT​TCC	*Pa*LOX2fw	CAA​GTG​GAG​CCG​TAG​ATA​AAC​TG
pBSY3Z_fw	GAA​ATT​CAC​CAT​AAC​ACT​TGC​TCT​AGT​C	*Gm*LOX_alpha_fw	GGT​GTC​TCT​CTC​GAG​AAG​AGA​GAG​GCC​GAA​GCT​CAC​CAT​CAC​CAT​CAC​CAC​AC
pBSY3Z_rv	CTC​AGG​CAA​ATG​GCA​TTC​TGA​C	*Tom*LOX_alpha_fw	GGT​GTC​TCT​CTC​GAG​AAG​AGA​GAG​GCC​GAA​GCT​CAT​CAC​CAC​CAC​CAT​CAC​AA CTT​CAG​AGT​GCA​TCA​CAA​CTA​C
*Ps*LOX1rv	GTT​CAC​GGA​AAT​GTC​TAA​TGG​GTT​G	*Ps*LOX1rv	GTT​CAC​GGA​AAT​GTC​TAA​TGG​GTT​G
*Ps*LOX2fw	CAA​CAA​GAG​AAC​CAA​CCC​ATT​AGA​C	*Fox*LOX_alpha_fw	GGT​GTC​TCT​CTC​GAG​AAG​AGA​GAG​GCC​GAA​GCT​CAT​CAT​CAC​CAT​CAC​CAC​CTG
*Gm*LOX1rv	GGA​ACT​CTT​GCA​ATC​TTC​TGA​TAA​CG	*Ps*LOX_alpha_fw	GGT​GTC​TCT​CTC​GAG​AAG​AGA​GAG​GCC​GAA​GCT​CAC​CAT​CAC​CAC​CAT​CAC​GTT​C
*Gm*LOX2fw	GTG​AAC​CCT​AAC​GTT​ATC​AGA​AGA​TTG	*Pa*LOX_alpha_fw	GGT​GTC​TCT​CTC​GAG​AAG​AGA​GAG​GCC​GAA​GCT​CAC​CAT​CAT​CAC​CAT​CAT​AAC​GAT​TC
*Tom*LOX1rv	CTA​ATT​GAG​TAA​GGG​TTC​AAA​CCA​GC	*Np*LOX_alpha_fw	GGT​GTC​TCT​CTC​GAG​AAG​AGA​GAG​GCC​GAA​GCT​CAT​CAC​CAC​CAT​CAC​CAT​AAG​C
*Tom*LOX2fw	GAC​AGA​CTT​TGG​CTG​GTT​TGA​AC	*Cl*HPL_alpha_fw	GGT​GTC​TCT​CTC​GAG​AAG​AGA​GAG​GCC​GAA​GCT​CAC​CAT​CAC​CAC​CAT​CAC​AAA​G
*Fox*LOX1rv	CAT​TAG​GTT​CGT​CCA​ATG​GAG​TG	*Mt*HPL_alpha_fw	GGT​GTC​TCT​CTC​GAG​AAG​AGA​GAG​GCC​GAA​GCT​CAC​CAC​CAT​CAC​CAC​CAT​GC
*Fox*LOX2fw	CTA​ACT​TGA​CCT​ACA​CTC​CAT​TGG	*Ms*HPL_alpha_fw	GGT​GTC​TCT​CTC​GAG​AAG​AGA​GAG​GCC​GAA​GCT​CAC​CAC​CAT​CAC​CAT​CAC​TTG

A Co-transformant of *Ps*LOX and *Mt*HPL was prepared as follows: A functional *Mt*HPL clone was selected after transformation of electrocompetent *K. phaffii* with *Swa*I linearized pBSY3Z_*Mt*HPL. This strain was made competent and transformed with *Swa*I linearized pBSY3Z_*Ps*LOX vector to obtain genomic integration of the expression cassette by homologous recombination.

Different combinations of promotors, inducible as well as constitutive, were tested (Table 3). These bidirectional promoter constructs were assembled by Gibson cloning using pBSY3Z_*Ps*LOX as the backbone. This strategy enables easy exchange of the bidirectional promoter system to minimize cloning efforts. The bidirectional promoters (Vogl et al., 2018a) were commercially available from bisy GmbH, Hofstätten an der Raab, Austria (https://www.bisy.at/#products, accessed 02.12.2022). Exchange of promoters was conducted *via* PCR. All primers used are listed in Table 1 and were ordered from Integrated DNA Technologies (IDT; San Jose, United States). Finally, backbones and bidirectional promoters were fused by Gibson assembly (Gibson et al., 2009) and were used to transform *E. coli* NEB5α (NEB, Frankfurt am Main, Germany). *Ps*LOX, *Ms*HPL and *Mt*HPL (Table 3) sequences were implemented as codon optimized genes (*Komagataella phaffii/Pichia pastoris* codon table). All assembled vectors were confirmed by sequencing (Microsynth; Balgach, Switzerland). The *K. phaffii* strain CBS7435 Mut^S^ (Küberl et al., 2011) was used as wild type host strain (WT) and as a negative control. Electrocompetent cells were transformed with 1 µg of *Swa*I linearized plasmids according to the protocol of Vogl et al. (2018b). Aliquots of transformed cells were plated on YPD agar containing 100 μg ml^−1^ of Zeocin™.

### Cultivation and expression

Cultivation and expression procedures were conducted as previously described (Hashem et al., 2020; Horvat et al., 2020). For each construct, approximately 80 clones were picked from selection plates. Cultivations were performed in 96 deep-well plates (DWP) in a volume of 500 µl. Clones were incubated in YPD medium at 28°C, at 320 rpm for 65 h. Each plate contained wells with *K. phaffii* WT strains, as negative controls and sterile controls. For larger scale cultivations, 50 ml media were inoculated from a 10 ml preculture in YPD to 2 OD_600_ units in 300 ml baffled shake flasks. Main cultures were started and induced with either BMGY (1% yeast extract, 2% peptone, 100 mM potassium phosphate, pH 6.0, 1.34% YNB, 4 × 10^-5^% biotin, 10 mM MgSO_4_ and 1% glycerol) or BMMY (1% yeast extract, 2% peptone, 100 mM potassium phosphate, pH 6.0, 1.34% YNB, 4 × 10^-5^% biotin, 10 mM MgSO_4_ and 1% methanol) at 28°C and 120 rpm for 48 h. Methanol and glycerol were added every 12 h to reach a concentration of 0.5% and 1%, respectively. OD_600_ measurements in microtiter plates were conducted in a Synergy Mx Plate reader (BioTek, Winooski, United States). *K. phaffii* cells were harvested by centrifugation at 4,000 rpm (3,220 × g) for 10 min in an Eppendorf tabletop 5810R centrifuge. Harvested cells were washed with McIlvain citrate-phosphate buffer, pH 6.5 (0.1 M citric acid, 0.2 M Na_2_HPO_4_), and cell pellets were stored at −20°C for subsequent bioconversions.

### Nickel affinity chromatography

Cells producing LOX and HPL intracellularly were harvested and resuspended in lysis buffer (100 mM Tris-HCl, pH 7.5, containing 1 mM EDTA and 2 mM PMSF). Equal volume of glass beads (0.5 mm, Sigma Aldrich) was added to the cells. After agitating the suspension 8 times for 30 s and chilling for 30 s on ice, the samples were centrifuged at 16,100 × *g* for 10 min at 4°C. Proteins were purified by nickel affinity chromatography using the gravity flow protocol for Ni-Sepharose 6 Fast Flow columns by GE Healthcare. The column was equilibrated with 10 column volumes (CV) of binding buffer (50 mM NaH_2_PO_4_, pH 7.0, 300 mM NaCl, and 10 mM imidazole). Cell free extracts as well as extracellularly produced enzymes secreted to 50 ml *K. phaffii* culture supernatants were applied to the column. The column was washed with binding and washing buffers, supplemented with imidazol to a final concentration of 30 mM and was eluted with elution buffer (50 mM NaH_2_PO_4_, pH 7.0, 300 mM NaCl, and 300 mM imidazole). Fractions that contained protein were pooled and concentrated to a volume of ∼ 2.5 ml. Buffer exchange was then performed *via* size exclusion chromatography using PD-10 columns (GE Healthcare), and samples were collected in 200 µL aliquots and stored at −20°C. This column had been equilibrated with 100 mM of sodium-phosphate buffer, pH 7.4.

### Specific activities of lipoxygenases and hydroperoxide lyases

LOX and HPL activity was determined spectrophotometrically by monitoring increases or decreases in the absorbance at 234 nm, respectively, based on the accumulation of hydroperoxydiene and its conversion (Gökmen et al., 2002; Plagemann et al., 2013; Kelle et al., 2014). The experiments were carried out in 96-well UV-Star microtiter plates. Wells contained enzyme dissolved in McIlvaine citrate-phosphate buffer, pH 6.5. A 2 mM linoleic acid or HPODE solution was used to initiate the reaction. For detailed procedure see Hashem et al., 2020. Blank reactions without substrate and without enzyme were carried out in parallel. Absorbance at 234 nm was monitored using a plate reader (Synergy Mx, BioTek) at 30°C to measure specific activities. All experiments were performed in quadruplicates.

### Assay preparation for the production of green leaf volatiles

Washed and frozen whole cells were thawed at +4°C and resuspended in McIlvain buffer to reach an OD_600_ of 50. Linoleic acid (0.1–1 M in EtOH) was added to the reaction mixture to give final concentrations of 5–10 mM at approximately 5% v/v EtOH. Substrate pulses were given 2 times a day after 4 h, 24 h, 28 h, 48 h, and 52 h at 5 mM feeds with lower EtOH concentrations to keep the overall ethanol levels low. Bioconversions were incubated for either 1 h, 3 h, 80 h or 100 h, at 320 rpm or 120 rpm for reactions in DWP or shake flask, respectively. All reactions were performed as biological and technical triplicates. At selected time points, reactions were terminated by the addition of 50 µl of 6 M HCl. Afterwards, 500 µl of each bioconversion was vortexed for 2 min with the same volume of ethyl acetate to extract organic compounds. The organic phase was dried with Na_2_SO_4_ (approximately 10% (w/v)) prior to transferring 200 µl into glass vials for GC analysis.

### GC-FID analysis of green leaf volatiles

Reactions were analyzed on a Shimadzu 2010 Plus equipped with a flame ionization detector and a ZB-5 column (30 m, 0.25 µm, 0.32 mm, Agilent technologies). Sample aliquots of 1 μl were injected in split mode (split ratio 10:1). A rapid method for hexanol detection was used as an indicator for active enzymes in screening. Column temperature was increased from 90°C to 320°C at 30°C/min. For a more precise quantification of produced green leaf volatiles we applied a slower ramp. Initial temperature was 70°C, and the oven temperature was increased to 320°C at 20°C/min. Analytical standards for hexanal and hexanol, as well as internal standard tetradecane, were used to determine product concentrations. Quantitation was performed based on linear intrapolation of calibration curves with concentrations of standards ranging from 0.05 mM to 20 mM.

## Results and discussion

Green leaf volatiles (GLVs) are formed by consecutive action of lipoxygenase (LOX) and hydroperoxide lyase (HPL) upon mastication of fruits and vegetables. Herein, we present the co-expression of LOX and HPL genes under the control of several different promoter pairs in the yeast *K. phaffii*. Secretion of LOX was studied in our lab with the aim of generating pure biocatalysts. *Fusarium oxysporum Fox*LOX and *Pseudomonas aeruginosa Pa*LOX secretion into the culture supernatant of *K. phaffii* was confirmed previously (Hashem et al., 2020), and one of the questions addressed herein was whether HPLs can be produced extracellularly by *K. phaffii,* however, none of the three HPLs targeted to the extracellular environment was active in the culture supernatant (Table 2).

**TABLE 2:** Intracellular and extracellular production of different LOX and HPL enzymes and their specific activities in *K. phaffii*.

Organism			Intracellular		Extracellular		Accession nr.
			Expressed	Activity (U mg^−1^)	Expressed	Activity (U mg^−1^)	
LOX	*Glycine max*	*Gm*	✓	10	—	0	NP_001236153
	*Solanum lycopersicum*	*Tom*	—	0	—	0	AAB65766
	*Fusarium oxysporum*	*Fox*	—	0	✓	5	EGU80482
	*Pleurotus sapidus*	*Ps*	✓	76	—	0	CCV01581.1
	*Pseudomonas aeruginosa*	*Pa*	✓	7	✓	11	NP_249860.1
HPL	*Medicago truncatula*	*Mt*	✓	16	—	0	XP_003606860
	*Medicago sativa*	*Ms*	✓	2	—	0	CAB54847.1
	*Citrullus lanatus*	*Cl*	✓	1	—	0	AAU12570.1

Intracellular expression of almost all selected LOXs and HPLs was successful, and highest activities were observed for *P. sapidus Ps*LOX and *M. truncatula Mt*HPL, showing specific activities of 76 U/mg and 16 U/mg, respectively (Table 2).

A strain expressing functional *Mt*HPL (cytosolic) was transformed to produce *Ps*LOX, which had had the highest specific activity of all tested LOXs (Table 2). This construct is referred to as Co-transformant (Co). Preliminary assays with whole cells showed promising results, confirming the production of the GLVs hexanol and hexanal.

The successful application of enzymatic cascades depends on a balanced level of single enzymes to ensure a possibly smooth flux from substrate to product without the accumulation of intermediates. Based on the specific activities of LOXs and HPLs (Table 2), we hypothesized that highest possible expression levels of HPL might be beneficial, while a low to medium amount of LOX would perhaps suffice. To test this assumption, various inducible and constitutive promoters in bidirectional (bidi) promoter systems were applied (Table 3). The bidi promoter library allows to identify ideal ratios of both enzymes and administer their expression level in one host, in order to overcome the bottleneck represented by HPL enzymes (Gigot et al., 2010). While *Mt*HPL was selected as the most promising HPL for the promoter library, *Medicago sativa* HPL (*Ms*HPL) was also a promising enzyme. Therefore, the effect of one particular bidi promoter on catalyst efficiency was directly compared with two different HPLs.

**TABLE 3:** Co-expression constructs of LOX and HPL.

Construct	Promoter-*Ps*LOX	Promoter-*Mt*HPL	Promoter-*Ms*HPL
Co-transformant (Co)	PDC^a^ ^,^ ^d^	PDC	
1	PDC	GAP^b^ ^,^ ^c^	
2	GAP	PDC	
3	AOX^a^ ^,^ ^c^	PDC	
4	TEF^b^ ^,^ ^c^	GAP	
5	GAP	TEF	
6	GAP	—	PDC
7	PDP^a^ ^,^ ^e^	PDC	
8	PDC	PDP	
9	PDC	AOX	
Negative control (NC)	—	—	

^a^ Inducible Promoter.

^b^ Constitutive Promoter

Promoter strength

^c^ Strong.

^d^ Medium.

^e^ Weak.

**FIGURE 1:**
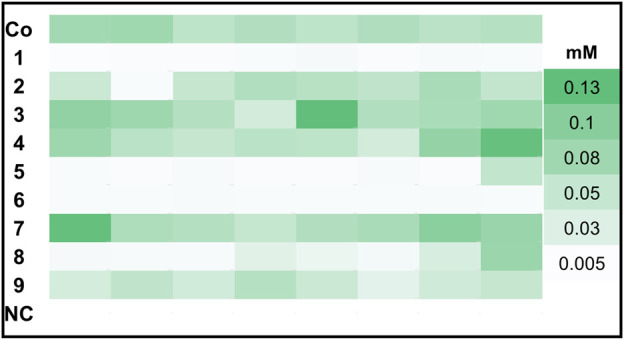
Hexanol production of top 8 clones harboring *Ps*LOX and *Mt*HPL or *Ms*HPL upon MeOH induction. *K. phaffii* WT, which was used as a negative control (NC) showed no production of hexanol.

In order to find the strains with highest GLV production we screened >86 clones per transformed construct for each promoter pair, because the integration locus and copy number influence biocatalyst activity. Hexanol is less volatile and reactive than hexanal and gave more reproducible analytical values in our hands. Therefore, hexanol formation was used as the reporter compound for GLV production in the initial GC-FID based screening of clone libraries. Prior to the whole cell assay, all constructs were cultivated in 96 DWPs and gene expression was induced with either methanol (MeOH) or glycerol (GY). The best eight clones per construct were rescreened. In Figure 1 we present an activity landscape of strains which were induced with MeOH, with the most active transformants shown in dark green and inactive transformants in white. Construct 3, 4 and 7 showed the highest hexanol concentration (0.13 mM) after incubating whole cells with linoleic acid (1 mM) for 1 h. The combination of *Ms*HPL in this context was not successful, as hardly any product could be detected for the clones of construct 6.

**FIGURE 2:**
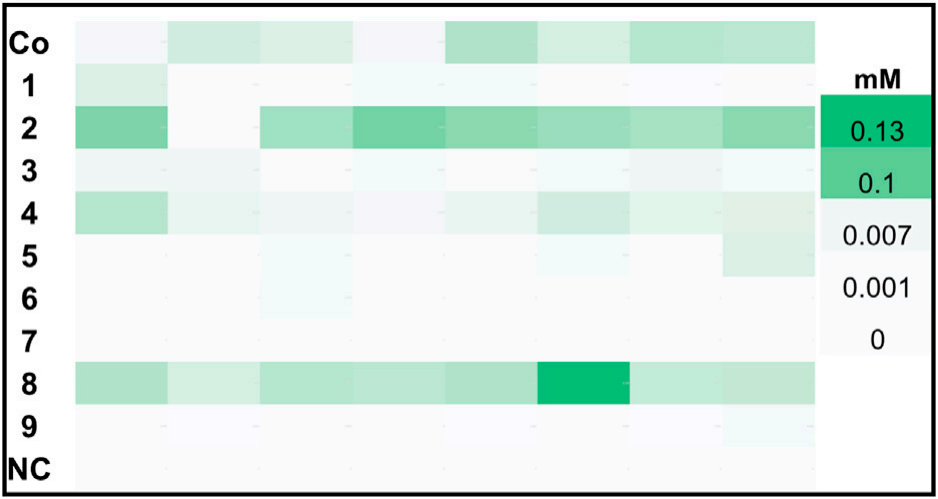
Hexanol production of top 8 clones harboring *Ps*LOX and *Mt*HPL or *Ms*HPL upon GY feed. *K. phaffii* WT, which was used as a negative control (NC) showed no production of hexanol.

Figure 2 shows a landscape of clones for the same constructs induced with glycerol. While construct 8, 2 and the Co-transformant showed the highest hexanol production under these conditions, a significant difference in the level of produced hexanol is obvious when compared to the MeOH induced constructs. Both MeOH and GY induced DWPs reached approximately the same cell densities, hence the activity per catalyst unit was higher for MeOH induced cells.

**FIGURE 3:**
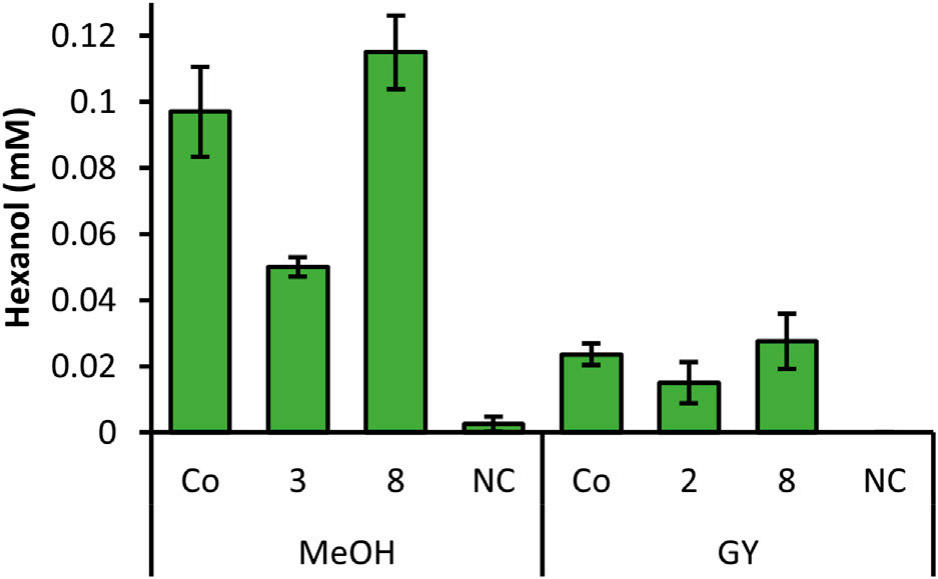
Summarized screening of selected strains from constructs with highest hexanol yield, cultivated in DWP in biological quadruplicates. Prior to whole cells assay, cells were induced with MeOH or GY. *K. phaffii* WT, which was used as a negative control (NC) showed no production of hexanol.

In order to investigate all constructs which showed promising results in the screening, we selected clones for each construct, which showed the highest hexanol level from Figures 1, 2 and cultivated these strains in quadruplicates with MeOH and GY (Figure 3). Out of all promising clones, Figure 3 shows the results with strains of construct 2, 3, 8 and the Co-transformant. These selected strains showed the highest and most reproducible production of hexanol, when cultivated in DWPs. The highest hexanol level were obtained when inducing construct 8 and Co-transformant with MeOH (Figure 3). However, upon GY induction a decent level of hexanol for the Co-transformant, construct 2 and 8 was obtained (Figure 3). Construct 8 with *Ps*LOX under the control of the medium strength PDC promoter and *Mt*HPL under the control of the weak, inducible PDP promoter is promising for both MeOH and glycerol induced intracellular production of the desired cascading enzymes.

**FIGURE 4:**
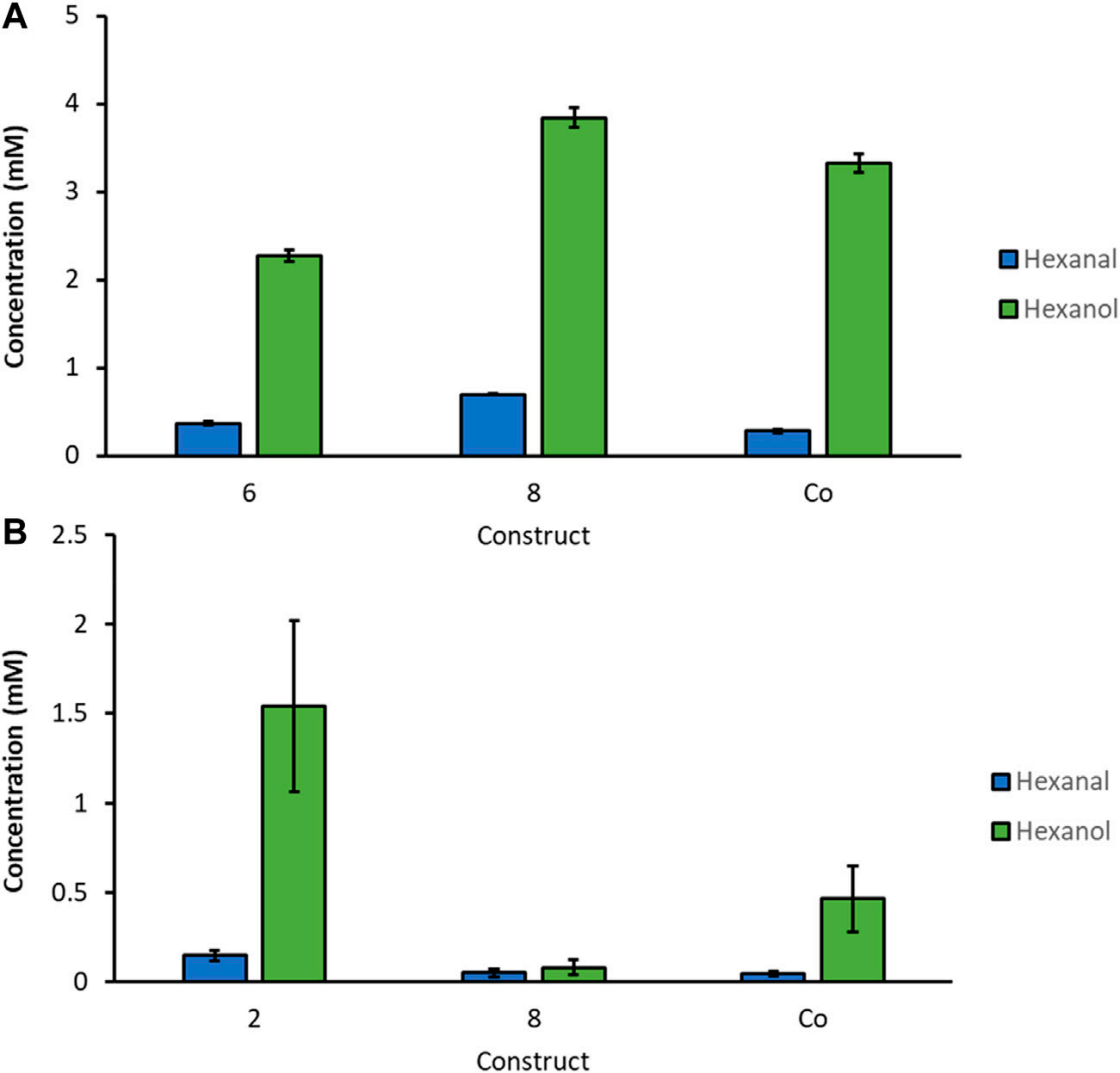
Biological replicates of most promising strains for selected constructs cultivated in baffled shaking flasks induced with **(A)**: MeOH and **(B)**: GY. 50 OD units of thawed whole cells were incubated with 10 mM linoleic acid for 3 h at 28°C.

By narrowing down the constructs to the specific clones with the highest hexanol levels we found in the screenings, we cultivated selected constructs in 3 biological replicates on a larger scale, in baffled shake flasks (300 ml). Protein expression was induced with MeOH or GY, respectively (Figures 4A,B). For activity assays, 50 OD units of freshly thawed whole cells were incubated with 10 mM of linoleic acid for 3 h in Pyrex tubes on a rotating platform at 28°C. Hexanol and hexanal production was quantified by GC-FID. We detected the highest GLV levels, approximately 4 mM, for construct 8 induced with MeOH, followed by the Co-transformant producing slightly less GLVs. GLV production upon GY induction was also promising. Construct 2 showed more than 1.5 mM hexanal and hexanol, after only 3 h of incubation.

**FIGURE 5:**
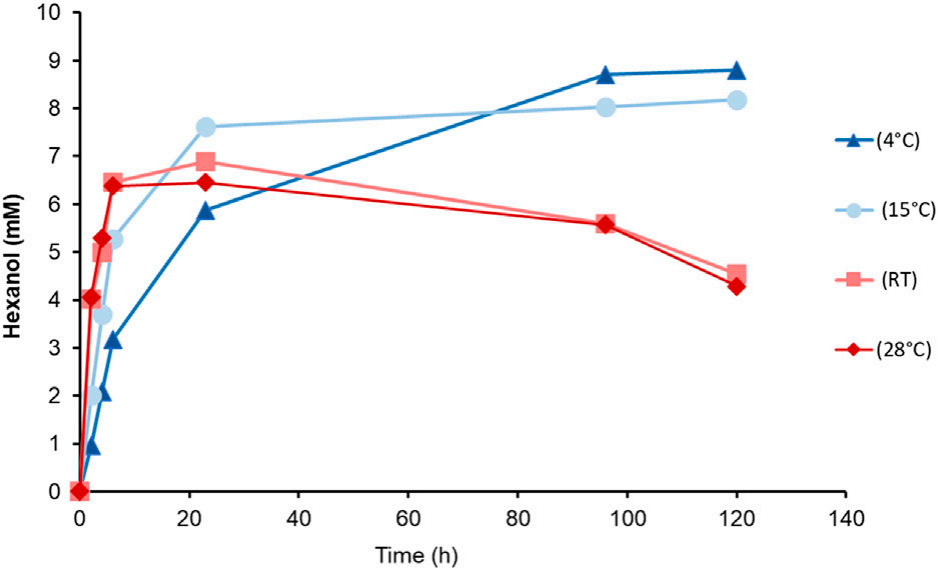
Time dependent hexanol formation upon incubation of *Ps*LOX and *Mt*HPL producing *K. phaffii* Co-transformant (MeOH) with linoleic acid at different temperatures.

In a long-term experiment, whole cells of the Co-transformant induced with MeOH, were incubated at different temperatures (Figure 5). We aimed to determine both the reaction rate and the recovery of produced GLV, in order to find the most suitable reaction temperature and reaction time. A substrate feed was implemented after 6 h, because of cellular consumption of linoleic acid. Although the highest detected level of hexanol was at 4°C, subsequent experiments were done at 15°C, which seemed to be a good compromise in terms of substrate solubility and product loss. Interestingly, more than 8 mM hexanol was detected after 100 h, reaching almost the same level as the samples incubated at 4°C. However, the initial product formation rate of cells incubated at 15°C was much higher, giving a higher yield in a shorter incubation period. Cells at 15°C showed fastest production of hexanol, reaching almost 8 mM after 23 h.

**FIGURE 6:**
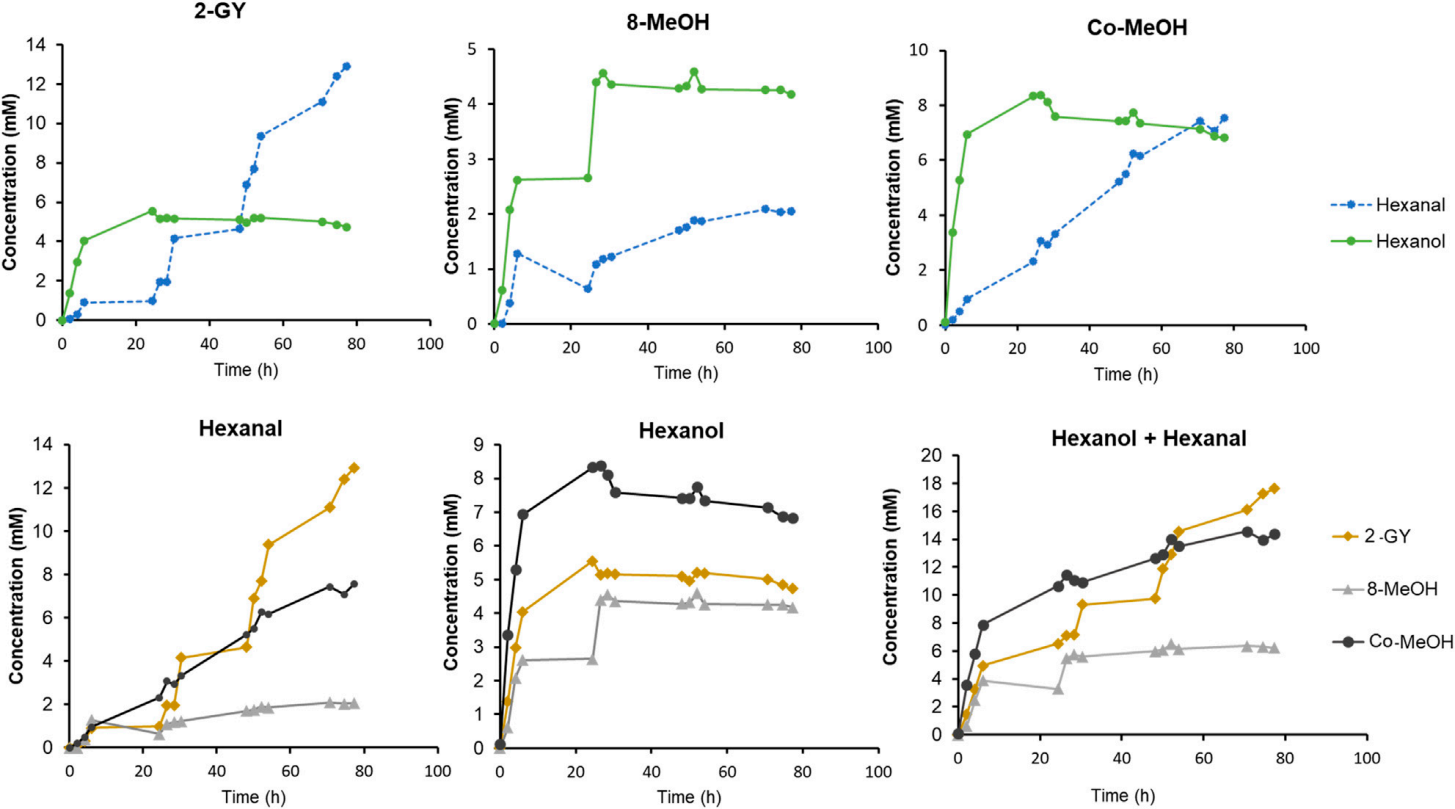
Time dependent GLV production by selected strains from construct 2, 8 and Co-transformant. Cells were induced by either GY or MeOH. Cultivation and conversions were conducted in baffled shake flask in a reaction volume of 25 ml, at 15°C and 10 mM linoleic acid, and subsequent substrate feed pulses.

Finally, GLV production by the best strains (i.e. construct 2, 8 and Co-transformant) was monitored over time. Therefore, *K. phaffii* whole cells were cultivated, *Ps*LOX and *Mt*HPL production was induced with either MeOH or GY, and the whole-cell biocatalysts were frozen, stored at −20°C and thawed before use for improved permeability (Chen, 2007; Abad et al., 2011; Rinnofner et al., 2019). All reactions were performed in baffled shake flasks with a reaction volume of approximately 25 ml for 80 h at 15°C (Figure 6). Subsequent substrate feed pulses were as specified in the Materials and Methods section.

After inducing construct 2 with GY, more than 5.5 mM hexanol was detected upon linoleic acid addition after 24 h. However, after some time, the hexanol level was slightly decreasing while the hexanal titre was increasing to up to 12.9 mM. This leads to an outstanding yield of GLVs, which reached almost 18 mM after 80 h. Compared to the work of Santiago-Gómez et al. (2009) we reached more than twice as much hexanal, while starting the reactions with the much cheaper linoleic acid, instead of the costly HPODE substrate.

For MeOH induced construct 8, approximately 5 mM of hexanol was observed after 24 h, and no further increase was observed within 80 h. In contrast, a slightly increasing level of hexanal was detected, reaching almost 2 mM.

The MeOH induced Co-transformant showed the highest hexanol production of all three constructs. Approximately 8 mM of hexanol was produced in 24 h. Afterwards, as hexanol level slightly decreased, an almost linear increase of hexanal was observed. This construct reached almost the same hexanal concentration as the level of hexanol after 80 h.

The faster production of hexanol in the first hours seem to appear in all constructs (Figure 6). As soon as hexanol production is stalling, the rise of hexanal concentration can be observed. The production of hexanal continues to increase while hexanol concentration slowly decreases. This leads to the assumption that in thawed cells reduction equivalents and/or reductases, involved in aldehyde metabolism, may be depleted after some time (O’Brien et al., 2005; Lopachin and Gavin, 2014; Laskar and Younus, 2019; Hu et al., 2020). Therefore, hexanal can no longer be reduced and accumulation thereof is noticed.

Although hexanol levels are not as high as presented in the work of Brühlmann and Bosijokovic, our system allows a simple access to GLVs, in terms of a preferable starting material as well as the possibility to select constructs that produce either high hexanol or hexanal levels (Brühlmann and Bosijokovic, 2016). Our system allowed for the generation of 10–20 times more GLVs, without a notable loss of activity even after days of biotransformation as compared to literature (Buchhaupt et al., 2012).

Cells cultivated and induced with GY proliferate for longer time than their analogues induced with MeOH. Due to the fact that GY induced construct 2 can reach cell densities that are approximately three times higher, it seems to be of particular interest for future applications. The ability to produce as much or even more GLVs with glycerol than with the (in)famous methanol induction of *K. phaffii,* makes our construct 2 the optimal candidate and a game changer for the production of natural quality GLVs.

## Conclusion

The combination of promoters with different promoter strength for co-expression of LOX and HPL suggested that the strength of a promoter is not of primary importance. Although strong promoters were used in almost all constructs, the level of produced GLVs could not be more diverse. More relevant appeared to be the presence of an inducible promoter upstream of the *HPL* gene, as in the case of constructs 2, 8 and the Co-transformant, whereas the promoter regulating LOX production did not influence product titers significantly. Furthermore, our system is producing higher product titers using glycerol as a carbon source. A LOX/HPL co-expression strain allows GLV production from the PUFAs, instead of the expensive and unstable hydroperoxides. These aspects are particularly important for industries in terms of an environmental, health and economic point of view. Our approach facilitated the expression of both enzymes in yeast host cells and, on top of that, counteracted other potential bottlenecks such as substrate inhibition, product toxicity and volatility.

## Data Availability

The original contributions presented in the study are included in the article; further inquiries can be directed to the corresponding author.
